# Safety and efficacy of the pipeline vantage flow diverter in small cerebral vessels ≤2.5 mm: a case series

**DOI:** 10.1007/s00234-026-03915-y

**Published:** 2026-01-31

**Authors:** Kaishin Tanaka, Alice Ma, Kenneth Faulder, Timothy Harrington, Brendan Steinfort

**Affiliations:** Royal North Shore Hospital, St Leonards, Australia

**Keywords:** Flow diverting stent, Pipeline embolization device, Cerebral aneurysm stenting, Small cerebral vessel, Aneurysm treatment

## Abstract

**Background:**

The Pipeline Vantage Flow Diverter (PVFD) with shield technology is the fourth and latest generation of the Pipeline embolization devices which are flow diverting stents used in the treatment of cerebral aneurysms. In early 2025 the larger PVFD 027s were recalled due to concerns of braid deformities with updated instructions provided for the PVFD 021. This study aimed at evaluating the safety and efficacy of the PVFD for cerebral aneurysm treatment in small parent vessels ≤ 2.5 mm.

**Methods:**

A retrospective review of patients who received a 2.5 mm diameter PVFD for cerebral aneurysm treatment at our institution over 4 years was performed. Baseline patient characteristics, complications, clinical and radiological outcomes were collected for each patient with a mean follow up duration of 1 year and 17 days (median 7 months and 5 days).

**Results:**

There were a total of 14 patients identified with a total of 16 aneurysms treated. At latest follow up complete aneurysm occlusion was achieved in 10 (66.7%) out of 16 treated aneurysms with 1 patient still awaiting follow up imaging. There were 2 (14.3%) of 14 patients who had immediate neurological complications, of which 1 was a transient deficit, and 0 patients with delayed complications. In-stent stenosis was found in 2 (15.4%) of 13 patients and asymptomatic stent occlusion occurred in 1 patient (7.69%).

**Conclusions:**

This case series demonstrated an acceptable safety profile with reasonable aneurysm occlusion rates for the use of PVFD in cerebral vessels ≤ 2.5 mm in treating aneurysms.

## Introduction

Flow diversion stenting (FDS) of intracranial aneurysms has become a well-established endovascular treatment modality [1]. The majority of the evidence details FDS use in the internal carotid arteries (ICA) and proximal branches of the circle of willis with studies showing its use in smaller and more distal vessels being more recent [2–4]. FDS of small cerebral vessels poses challenges not only due to the more difficult anatomy and more acute branching angles of distal vessels but also concerns for increased thrombogenicity due to the lower flow rates and increased exposed cross sectional metal surface area [4, 5]. Refinement and improvement of flow diverter technology over the years has alleviated these barriers [6].

The Pipeline Vantage Flow Diverter™ (PVFD) (Medtronic, Irvine, CA) is the latest generation of the Pipeline Embolization Device (PED) line spanning back three previous generations of devices. The PVFD improves on its predecessors with thinner wire diameters which results in an overall decreased device wall thickness [7, 8]. This allows for improved device visibility, ease of deployment, recapture and increased pore density [9]. There are a range of stent diameters with the largest measuring at 6 mm and the smallest being 2.5 mm. Diameters ≤ 3.5 mm are deployed by means of a 0.021 inch microcatheter (PVFD 021) and those that are ≥ 3.5 mm are designed to be deployed with a 0.027 inch microcatheter (PVFD 027). Several case series and studies have detailed the general use of the PVFD and its associated outcomes overall demonstrating adequate device performance, ease of use and favorable aneurysm occlusion rates [7–11]. The larger PVFD 027 s were recalled in early 2025 due to concerns of braid deformities, namely “fishmouthing” and incomplete stent wall apposition with updated instructions released for the PVFD 021 stents [12]. To date, the PVFD 021 performance in small cerebral vessels (≤ 2.5 mm diameter) has not been specifically evaluated. This single centre case series aimed to evaluate the feasibility, safety, clinical and radiological outcomes of the PVFD in treatment of cerebral aneurysms in small cerebral vessels ≤ 2.5 mm diameter.

## Methods

A retrospective review of a database of patients who had insertion of a fourth generation Pipeline Vantage flow diverting stent for treatment of intracranial aneurysms was performed from the dates of January 2021 to May 2025. We then identified the patients who had 2.5 mm stent diameter stents inserted. Patient characteristics, aneurysm characteristics, parent vessel sizes and use of adjunctive coiling or angioplasty was collected. Data was sourced from electronic medical records, electronic radiology images and procedure reports. Clinical follow up in each patient was performed 1 month post intervention with progress imaging performed at about 3 months. Initial progress imaging consisted of either a CTA when coiling was not utilized or an MRA. A cerebral DSA was then usually performed at about 6–12 months unless there was an indication for it to be performed earlier as determined at progress reviews. Subsequent follow up reviews extended up to 3 years and 1 month at the longest to date (Mean duration of 1 year 17 days, range from 1 month to 3 years and 1 month) dependent on each patient’s progress.

Neurological complications were classified as immediate (including intraprocedural complications within 24 h) and delayed (beyond 24 h). Any clinical neurological deficit or neuroradiological complications (hemorrhagic, ischemic or device related) due to the stenting regardless of severity were screened for and categorized into these subgroups. Aneurysm occlusion classification was assigned according to the O’Kelly-Marotta grading scale based off of each patient’s latest available progress imaging. There is 1 patient within this study who has had their 1 month clinical follow up but is currently awaiting their progress imaging at the time of this study thus they have not been assigned an aneurysm occlusion class. This patient was included to provide data on early and delayed complication rates.

This study was approved by our institution’s patient safety and quality unit. Given the small patient numbers, retrospective nature of the study and the absence of any identifying data, this study was deemed as not requiring ethics approval by our institution’s research and governance office and patient consent to participate was waived.

## Procedure

Procedures were all performed under general anaesthesia. All planned cases were commenced on aspirin 100 mg daily and prasugrel 10 mg (5 mg if body weight was less than 60 kg) daily 5 days prior to the procedure date. In emergency cases associated with acute subarachnoid haemorrhage from aneurysm rupture patients were loaded with prasugrel 30 mg peri-operatively and IV aspirin 500 mg intraoperatively. Intravenous heparin was administered intraoperatively aiming for an ACT at least 2 times the upper limit of normal with further heparin given hourly if required to maintain the ACT. Post procedure a heparin infusion of 500 units per hour was continued for 12–24 h.

The procedures were all performed using femoral access. Either a biaxial or triaxial access system was used depending on patient anatomy and interventionalist preference. PVFD stents 2.5 mm in diameter were all deployed utilizing 0.021 inch Phenom 21 microcatheters.

Dual antiplatelet therapy was continued for at least 3 months and then patients were continued on aspirin as a single agent. Dual antiplatelet therapy was extended beyond 3 months in some cases when the interventionalist deemed a clinical requirement for this, typically for persistent in-stent stenosis.

## Results

There were a total of 118 patients who underwent insertion of a flow diverting stent for treatment of an intracranial aneurysm at our institution within the specified period. Of this group, 14 patients were identified who had one or more 2.5 mm diameter Pipeline Vantage flow diverting stents inserted. There were 3 cases performed for emergent aneurysm rupture and the remaining 11 were performed electively. A total of 16 aneurysms were treated and there were 2 that had previously been coiled. Eleven (94.1%) of patients were female and the median age was 68 years (IQR 56–72 years). The mean aneurysm size was 6.19 mm (range from 0.7 to 18 mm) with 14 aneurysms (85.7%) in the anterior circulation and 2 (14.3%) in the posterior inferior cerebellar artery of the posterior circulation. The mean proximal vessel diameter was 1.73 mm (range 1.30–2.80 mm) and the mean distal vessel diameter was 1.49 mm (range 0.9–2.30 mm). There were 2 patients that had adjunctive coiling, one was for treatment of a ruptured aneurysm to assist with hemostasis, and the other was for treatment of an unruptured residual aneurysm neck in a previously coiled aneurysm. Adjunctive coiling was utilized in the second patient to ensure optimal aneurysmal occlusion due to its proximity to a fenestration complex of the anterior communicating artery that would complicate stent placement. Both aneurysms were located in the anterior circulation. There were 2 patients who each had 2 stents placed due to suboptimal positioning of the first stent over the aneurysm necks. These patients also had adjunctive balloon angioplasty performed to improve apposition of the two stents placed within each other. The details of baseline patient and aneurysm characteristics are seen in Table 1.

**Table 1:** Baseline patient characteristics. (ICA – internal carotid artery, MCA – Middle cerebral artery, ACOM – anterior communication artery, ACA – anterior cerebral artery, PICA – posterior inferior cerebellar artery)

Characteristic	Value (*N* = 14)
Age (years)	
Median (interquartile range)	68 (56–72)
Range	52–77
Gender (%)	
Female	11 (78.57)
Male	3 (21.43)
Baseline mRS (%)	
0	11 (78.6)
1	3 (21.4)
Mean (range)	0 (0–1)
Past medical history (%)	
Hypertension	10 (71.4)
Hypercholesterolaemia	7 (50.0)
Diabetes mellitus	1 (7.14)
Parent vessel diameters (mm)	
Proximal vessel mean (range)	1.73 (1.30–2.80)
Distal vessel mean (range)	1.49 (0.90–2.30)
Aneurysm size (mm)	
Number of treated aneurysms	16
Number previously coiled	2
Mean size (range)	6.19 (0.7–18)
Aneurysm location (%)	
Anterior circulation	14 (87.5)
ICA (Ophthalmic)	1 (6.25)
MCA (M1 – M2)	2 (12.5)
ACOM (A1 – A2)	4 (25.0)
ACA (A2 – A3)	7 (43.8)
Posterior circulation (PICA)	2 (12.5)
Adjunctive intervention (%)	
Coiling	2 (14.3)
Balloon angioplasty	2 (14.3)

One patient developed transient hemiparesis within 24 h of stent insertion which fully resolved within a few hours. The patient was given a bolus dose of abciximab and a repeat DSA demonstrated patency of the stent with no thrombus formation. One patient suffered from a fisher grade 4 subarachnoid haemorrhage within 24 h of stent insertion. This was an elective case that was uncomplicated with gross iatrogenic vessel injury excluded on post-procedural DSA runs. A subsequent repeat DSA revealed no vessel wall injury, no active haemorrhage from any aneurysm and adequate stent patency. A clear cause for the subarachnoid haemorrhage for this patient was not found. This is consequently the single patient that ended up with a mRS of 4 at the latest follow up review.

The remaining patients had no early or delayed neurological complications related to the stent at their latest follow up. There was one patient who was treated for acute aneurysm rupture with subarachnoid haemorrhage and went from an mRS of 0 to 1 at follow up. This was due to the development of hydrocephalus requiring the insertion of a ventriculoperitoneal shunt, a known consequence of subarachnoid haemorrhage and not directly related to the stent insertion. The remainder of the patients remained an mRS of 0 at latest follow up.

**Fig. 1:**
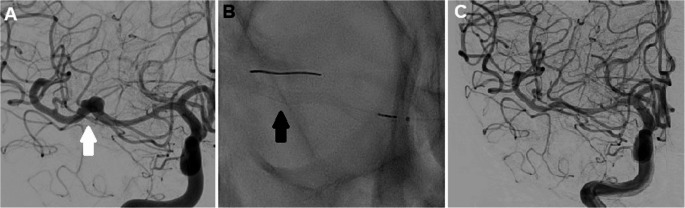
**A.**DSA, right internal carotid injection, anterior view. Fusiform aneurysm arising from the right M1 (*white arrow*)**B.** PVFD 2.5 x 20mm insertion (*black arrow*)**C.** DSA, right internal carotid injection, anterior view. Complete occlusion of the aneurysm achieved at 1 year

**Fig. 2:**
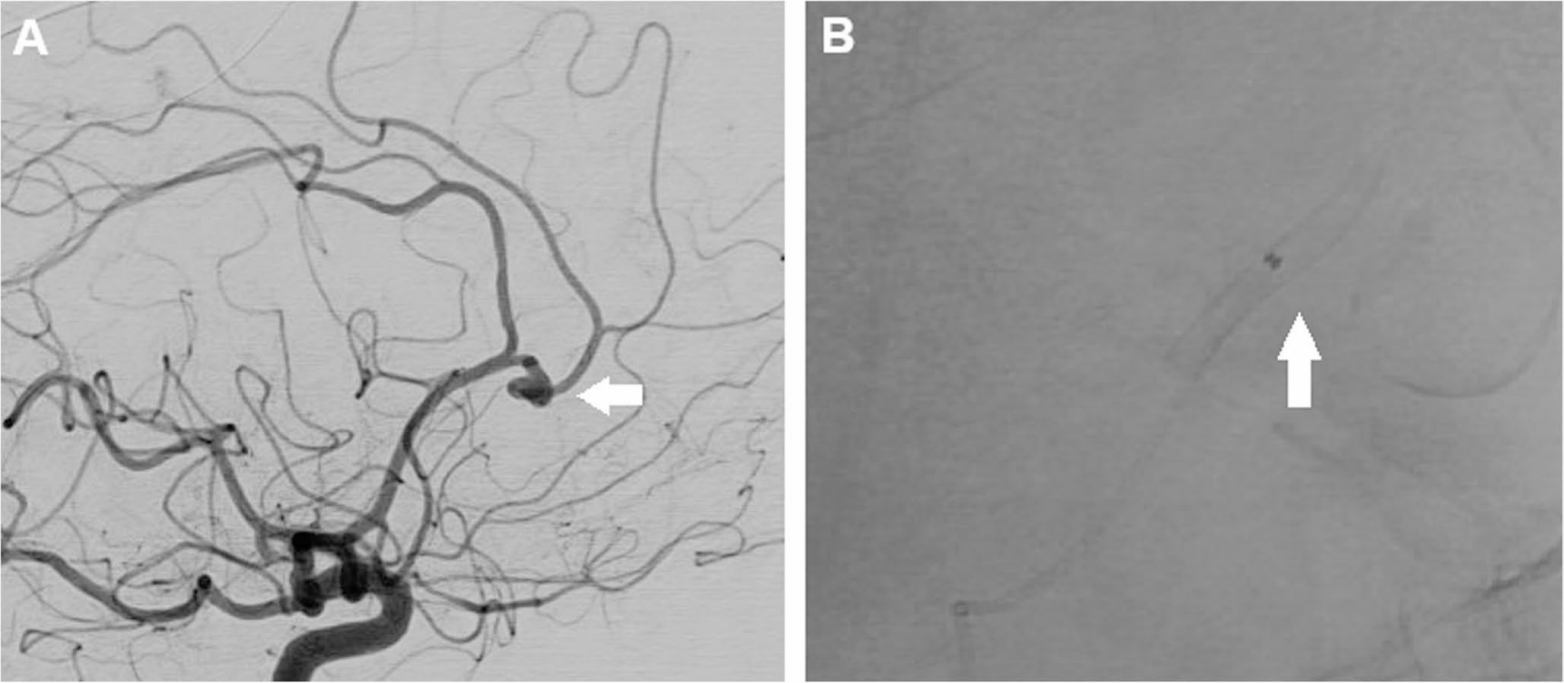
**A. **DSA, right internal carotid injection, lateral view. Aneurysm arising from the callosomarginal artery with a “Z” shaped bend (*arrow*).**B**. PVFD 2.5 x 12mm inserted into the adjacent pericallosal artery (*arrow)*

Aneurysm occlusion class was assigned using the O’Kelly-Marotta grading scale based on each patient’s latest follow up imaging. There was 1 patient who had not yet had follow up imaging at the time of writing and thus could not be assigned an occlusion class. There were 10 aneurysms (66.7%) that achieved grade D occlusion (full aneurysmal occlusion), 2 aneurysms (13.3%) with grade C2 (neck remnant seen in the capillary phase), 2 aneurysms with grade B2 (subtotal aneurysmal filling seen in the capillary phase) and 1 aneurysm (6.67%) with grade A1 (residual total filling aneurysm in arterial phase) (Fig. 1). The patient with grade A1 occlusion originally had an aneurysm arising from the origin of the callosomarginal artery with an acute “Z” shaped angle coming off the parent A2 vessel (Fig. 2). PVFD stent placement directly across the artery was therefore not technically possible and after a multidisciplinary discussion it was decided to place the stent in the pericallosal artery instead to provide some flow diverting effect. At latest follow up, despite ongoing arterial phase aneurysmal filling there was some noted decrease in overall aneurysmal dimensions. This particular patient also had in-stent stenosis, likely contributed by the patient’s variable compliance with oral anti-platelet agents. There was 1 patient who had an asymptomatic stent occlusion with follow up DSA demonstrating pial collateralization of the distal vessel branches. There were no access site related complications in any patient in this study. Clinical and radiological outcomes are summarized in Table 2.

**Table 2:** Clinical outcomes

Characteristic	Frequency (*N* = 14)
Immediate neurological complications (%)	2 (14.3)
Transient neurological deficit	1 (7.14)
Hemorrhagic	1 (7.14)
Delayed neurological complications (%)	0 (0)
Latest follow up duration	
Mean	1 year, 17 days
Median (IQR)	7 months, 5 days (5 months – 30 months)
Range	1 month – 3 years, 1 month
mRS at latest follow up (%)	
0	10 (71.4)
1	3 (21.4)
4	1 (7.14)
Mean (range)	1 (0–4)
Aneurysm occlusion grade at follow up (%)*	
(Data not available for 1 aneurysm)	
Grade D	10 (66.7)
Grade C	2 (13.3)
Grade B	2 (13.3)
Grade A	1 (6.67)
In-stent stenosis (%) (Data not available for 1 patient)	2 (15.4)
Stent occlusion (%)	
(Data not available for 1 patient)	1 (7.69)

*Using the O’Kelly-Marotta grading scale, 15 aneurysms in total

## Discussion

This retrospective observational case series demonstrates reasonable aneurysm occlusion rates in the use of the fourth generation PVFD 021 in small cerebral vessels ≤ 2.5 mm in diameter achieving 66.7% total occlusion and 13.3% grade C occlusion with a combined occlusion rate of 80.0%. There was an acceptable safety profile observed with a 14.3% rate of immediate complications, one of which was a transient deficit, and no delayed complications seen up to a median follow up duration of 7 months and 5 days. Thrombotic and haemorrhagic complication rates in this study were comparable to prior studies of FDS in small vessels with a single case of post procedural subarachnoid hemorrhage and a single case of asymptomatic stent occlusion. The overall safety, complication and efficacy profile of the PVFD 021 in small vessels observed in this study is in keeping with the previously published initial reports on its general use in a wide range of vessel sizes [8–11]. Braid deformities that have been seen at higher rates in the larger PVFD 027 were not observed in the PVFD 021 use in small vessels [12]. This case series provides incremental data specific to the PVFD 021 in small vessels and further studies are needed to expand on the limited conclusions that can be drawn from this sample size.

Our study found a relatively similar complication rate with high implantation success of the PVFD in small vessels as has previously been described in studies of older generation stents and other flow diverting devices [2, 6, 13, 14]. Smaller vessel stent placement demands finer technical navigation within typically more distal cerebral vessels to avoid vessel perforation, dissection or wire injury. In our study, there was 1 patient (7.14%) who suffered from an early post-procedural subarachnoid haemorrhage although repeat DSAs and other diagnostic imaging did not identify a perforation or other clear source of haemorrhage. The technical placement of the stent was uncomplicated and certainly this has been the experience at our institution overall for the placement of the PVFD is small vessels. Given diagnostic imaging did not reveal a clear cause, we speculate the haemorrhage may have been in part due to a minor iatrogenic vessel injury not seen on DSA in combination with a supratherapeutic post-intervention heparin dosing as this patient retrospectively had a below average body weight. Aneurysm rupture was deemed a less likely cause given the stable appearance on post-procedural and repeat DSAs however it is difficult to definitively determine the etiology of this complication. The overall rate of neurological morbidity for flow diverting stent placement is about 4.5% as previously reported in a meta-analysis by Zhou et al. [15] however the isolated haemorrhagic complication rate for FDS in small vessels may be slightly higher ranging from 3.36% to 7.40% as reported in a recent meta-analysis by Elek et al. [16]. The hemorrhagic complication rate of 7.14% (Table 2) in this series is in a similar range to these studies.

Asides from the clear increased navigational difficulty of stent placement in a small vessel, we found that the delivery of the PVFD stents to vessels of this size to be of comparable technical difficulty with the benefit of improved maneuverability afforded by the smaller 0.021” microcatheters compared to previous generation stents. There were also no vessel dissection or wire injuries encountered in this study.

The thrombotic complication rate in this study was found to be at a similar level to previous reports in the literature with one patient developing asymptomatic stent occlusion and one patient with a transient neurological deficit that we hypothesize to have been a minor thrombotic event giving a total rate of 14.3% (Table 2). The overall thrombotic complication rate for PEDs previously reported in the literature ranges from 4% to 9% [17, 18] with the recent meta-analysis by Elek et al. reporting a range of 8.5% − 16.1% specifically in small vessels [16]. The main caveat to this comparison is the small sample size of this study which limits the conclusions that can be drawn. Flow diversion stent placement in small vessels carries greater treatment related thromboembolic complications than larger vessels due to the lower flow rates and increased exposure of metal per cross-sectional stent area [6]. The PVFD is composed of smaller diameter wires compared to its predecessor, and this reduces the device thickness which therefore reduces the circulation exposed cross-sectional stent area. In our study, we suspect that the 1 patient who had an immediate post-procedural hemiparesis despite a repeat DSA (post empirical administration of abciximab) demonstrating in-stent patency may have had a transient stent thrombosis with rapid response to abciximab. This patient subsequently developed in-stent stenosis at latest follow up however this also likely occurred due to the patient’s variable compliance with antiplatelet medication. Although device thrombogenicity can be reduced by improved engineering, the risk is not eliminated, and it remains crucial for patients to adhere to antiplatelet medication. The overall in-stent stenosis rate determined in this study was likely confounded by this patient and may be an over estimation of the true rate.

The patient who developed an asymptomatic total stent occlusion had this found incidentally 4 months after stent placement on routine follow up imaging. This patient was compliant with antiplatelets and on repeat DSA was shown to have well-formed pial collaterals explaining why they remained asymptomatic. It is unclear as to why this occurred however asymptomatic stent occlusion has previously been reported and we hypothesize that it may be due to idiosyncratic anatomic or thrombotic effects affecting in-stent flow [8].

Overall, the clinical outcomes for the majority of patients was favorable with a mRS of 0 or 1 at latest follow up being achieved in 92.9% of patients. The only patient that had a change in mRS went from 0 to 4 at latest follow up. This occurred in the previously mentioned patient who suffered a subarachnoid haemorrhage. These results are comparable with other recent larger studies that evaluated the general use of the PVFD in addition to previous studies on earlier generation stents achieving favorable mRS scores of 0 − 1 with rates of 88–100% [6, 9]. Neurological morbidity rates in these studies range from 3% to 4.9% which is also comparable to our study with only the post-procedural subarachnoid patient having had a clinically significant change in mRS [8, 19].

In this study, the PVFD achieved complete aneurysm occlusion (Grade D) in 66.7% and near complete occlusion (Grade C) in 13.3% of the cases at latest follow up totaling to an adequate occlusion rate of 80.0% per the O’Kelly-Marotta grading scale (with follow up data still pending for 1 patient). It is again important to note the small sample size used to determine these occlusion rates in addition to the variability in follow up necessitates restraint in drawing conclusions from this data. A study by Hohenstatt et al. that reviewed the use of 5 different flow diverters in small cerebral vessels < 2 mm in diameter achieved comparable aneurysm occlusion rates to our study with a total reported occlusion rate of 55.4% and near-complete occlusion in 25% [6]. These occlusion rates are in fact lower than the overall reported rates for the PVFD analyzed in any vessel size with a study by de Velliers et al. achieving 94% and another study by Catala et al. achieving 97% overall total occlusion at 12 months [8, 10]. One factor that may explain this is that the deployment of 2.5 mm diameter stents in vessels smaller than 2.0 mm could lead to increased stent porosity due to suboptimal stent expansion and therefore a reduction in the flow diverting effect [20, 21]. The PVFD fortunately has a smaller stent wire diameter and overall higher pore density compared to previous generation flow diverters and this may play a role in maintaining flow diversion despite suboptimal stent expansion hence why the total occlusion rate achieved in this study was greater than that seen in Hohenstatt et al. [6]. Another factor leading to lower aneurysm occlusion rates that was noted in this study is the difficulty in achieving optimal stent placement in small vessels, particularly when the vascular anatomy becomes complex. The patient in this case series that had the aneurysm arising from the callosomarginal artery with an acute “Z” shaped angle from the parent A2 vessel at the latest follow up at 17 months only achieved a grade A1 occlusion class with marginal reduction in overall aneurysm size. This patient will require ongoing close follow-up to explore alternative endovascular or surgical treatment options. This case demonstrates how aneurysms located in small, distal vessels can be difficult to directly insert a flow diverting stent across which translates to overall lower aneurysm occlusion rates when alternative flow diverting strategies are employed. Malpositioned or FDS placement adjacent to the aneurysm neck has been shown in experimental haemodynamic modelling to still cause a reduction in intra-aneurysmal flow [22]. Furthermore, a retrospective multicenter study by Schob et al. demonstrated that indirect flow diversion can achieve aneurysmal occlusion in a subset of patients with a favourable short term safety profile [23].

To our knowledge, this study is the first study specifically assessing the use of the new generation PVFD in treating cerebral aneurysms in small cerebral vessels < 2.5 mm. This study is limited by the small sample size, its retrospective design and heterogeneity in patient characteristics and follow up approaches. The conclusions that can be drawn from this study are therefore limited in scope. Further prospective studies with large patient cohorts and standardized follow-up procedures are needed to build upon the findings of this study.

## Conclusion

This case series demonstrates that the use of the PVFD in small cerebral vessels ≤ 2.5 mm for the treatment of aneurysms had a reasonable safety profile with acceptable clinical and radiological outcomes. These results should be interpreted with caution given the small sample size. Larger studies are required to validate this and assess if occlusion rates may be lower compared to PVFD use in larger parent vessels.

## Data Availability

No datasets were generated or analysed during the current study.
